# Enhancing Photovoltaic Performance of Plasmonic Silicon Solar Cells with ITO Nanoparticles Dispersed in SiO_2_ Anti-Reflective Layer

**DOI:** 10.3390/ma12101614

**Published:** 2019-05-16

**Authors:** Wen-Jeng Ho, Guan-Yu Chen, Jheng-Jie Liu

**Affiliations:** Department of Electro-Optical Engineering, National Taipei University of Technology, No. 1, Section 3, Zhongxial East Road, Taipei 10608, Taiwan; a840112a@gmail.com (G.-Y.C.); jjliu@mail.ntut.edu.tw (J.-J.L.)

**Keywords:** ITO nanoparticles (ITO NPs), photovoltaic performance, plasmonic scattering, anti-reflective layer

## Abstract

In this study, we sought to enhance the photovoltaic performance of silicon solar cells by coating them (via the spin-on film technique) with a layer of SiO_2_ containing plasmonic indium-tin-oxide nanoparticles (ITO-NPs) of various concentrations. We demonstrated that the surface plasmon resonance absorption, surface morphology, and transmittance of the ITO-NPs dispersed in SiO_2_ layer at various concentrations (1–7 wt%). We also assessed the plasmonic scattering effects of ITO-NPs within a layer of SiO_2_ with and without a sub-layer of ITO in terms of optical reflectance, external quantum efficiency, and photovoltaic current-voltage under air mass (AM) 1.5G solar simulation. Compared to an uncoated reference silicon solar cell, applying a layer of SiO_2_ containing 3 wt% ITO-NPs improved efficiency by 17.90%, whereas applying the same layer over a sub-layer of ITO improved efficiency by 33.27%, due to the combined effects of anti-reflection and plasmonic scattering.

## 1. Introduction

Exploiting the immense potential of solar energy requires highly efficient photovoltaic devices. At present, wafer-based crystalline silicon is the dominant photovoltaic technology. Conversion efficiency and manufacturing expense are the two primary challenges to lowering the cost of electricity generated by photovoltaic systems. Silicon solar cells with a variety of nano- and micro-structures have been developed to enhance light trapping effects, reduce reflectance, and extend broadband performance. The plasmonic effects of nanoparticles (NPs) have also been applied to the problem of light trapping in solar cells [1,2,3,4]. Metallic nanoparticles exhibit strong optical extinction, due to the collective oscillation of free electrons, referred to as localized surface plasmon resonance (LSPR) [5,6]. Resonance wavelength is sensitive to the size and shape of both the NPs and the surrounding material [7]. When metal nanoparticles made of gold (Au NPs) [8,9,10], silver (Ag NPs) [11,12,13,14], and aluminium (Al NPs) [15,16,17] are used to scatter the light, more of the light energy can be trapped (to create highly efficient solar cells). Plasmonic nano-sized materials based on transparent oxide semiconductors, such as indium oxide (In_2_O_3_), zinc oxide (ZnO), and tin oxide (SnO_2_), have also been investigated in this regard [18,19,20]. Kanehara et al. reported that indium tin oxide (In_2_O_3_:Sn) nanoparticles (ITO-NPs) exhibited surface plasmon resonance (SPR) in the near-infrared region, with characteristics similar to those of noble metals [21]. In addition, Dhar et al. reported enhanced light trapping when ITO-NPs were embedded in the rear surface of a-Si/c-Si heterojunction solar cells [22,23,24]. However, relatively little research has been conducted to determine whether the distribution of ITO-NPs within a SiO_2_ anti-reflective layer can enhance the efficiency of silicon solar cells.

In this study, we sought to enhance the photovoltaic performance of silicon solar cells by depositing plasmonic anti-reflective layers on the surfaces of these cells via the spin-on film technique. The surface plasmon resonance absorption, surface morphology, and transmittance of the ITO NPs dispersed in SiO_2_ layer at various concentrations (1–7 wt%) were investigated by a Ultraviolet-Visible-Near-Infrared (UV-VIS-NIR) spectrophotometer and scanning electron microscope (SEM), respectively. Specifically, we created the silicon solar cells with two types of surface coatings: (1) Coatings which were comprised of a single layer of SiO_2_ and contained ITO-NPs embedded at various concentrations, and (2) compound surface coatings which comprised a layer of ITO beneath a layer of SiO_2_ with ITO-NPs embedded at various concentrations. We also examined the optical and electrical properties of cells with and without ITO-NPs in terms of dark current-voltage, optical reflectance and external quantum efficiency (EQE). Furthermore, we used photovoltaic current density-voltage (J-V) measurements to confirm the degree to which the various surface coatings enhanced photovoltaic performance. Finally, we evaluated the ability of surface coatings comprising ITO-NPs dispersed in a SiO_2_ layer to enhance the conversion efficiency of solar cells.

## 2. Experimental Methods 

### 2.1. Characterization of Optical Properties and Surface Morphology of the ITO Nanoparticles Dispersed in a SiO_2_ Layer

Prior to film deposition, quartz-glass substrates were ultrasonically cleaned in acetone, isopropanol and deionized water for 10 min, respectively, and were then dried with a flow of nitrogen. Four quartz-glass substrates were respectively coated with a SiO_2_ layer containing ITO-NPs at a concentration of 1, 3, 5, or 7 wt%. As a control, we prepared a quartz-glass substrate with the same SiO_2_ layer but without ITO-NPs for characterization comparing. The deposition solution comprised 1.94 g of silicate solution combined with ITO powder at a concentration of 1, 3, 5, or 7 wt%. The ITO powder used in this study was approximately between 20–70 nm in diameter (the information of particles size was provided by the vendor). The mixture was dropped on the clean quartz-glass substrates and held for 10 s before spin-coating, which was performed at 1200 rpm for 50 s. The deposited sample was subsequently baked at 200 °C for 5 min under an air atmosphere. The surface plasmon resonance absorption, surface morphology, and transmittance of the samples were measured with a UV-VIS-NIR spectrophotometer (Hitachi U-2800A, Hitachi High-Technologies Corporation, Tokyo, Japan) and SEM (Hitachi S-4700, Hitachi High-Tech Fielding Corporation). All measurements were performed at room temperature.

### 2.2. Fabrication and Characterization of Bare Silicon Solar Cells and Cells Coated with an Anti-Reflective Layer of SiO_2_ or ITO

**Figure 1:**
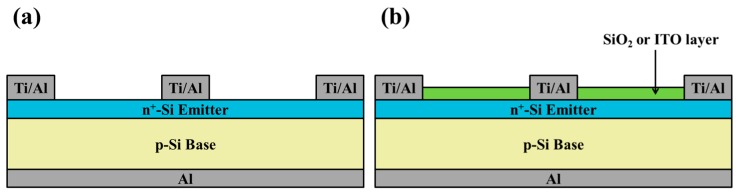
Schematic diagram of (**a**) the bare solar cell, (**b**) cell with SiO_2_ (250 nm) or ITO (54 nm) antireflective layer. ITO, indium-tin-oxide.

A (100)-oriented, P-type silicon wafer (boron-doped, thickness of 400 μm, and resistivity of 10 Ω-cm) was used as the base substrate for the solar cell devices. The wafer was cut into samples which measured 1 × 1 cm^2^ to produce bare solar cells. After standard cleaning, a 0.35-μm n^+^-silicon emitter layer was formed on the front surfaces of samples via spin-on film processing with a liquid phosphorous source. The samples were then subjected to heat treatment (900 °C) in a rapid thermal annealing (RTA) chamber under ambient N_2_ for 3 min. The oxide that remained on the surface of the silicon samples following diffusion was removed using a buffered oxide etchant (BOE). The n^+^-silicon emitter layer presented sheet resistance of 70 Ω/sq, as measured using a four-point probe resistivity system. We then deposited a 300-nm aluminum (Al) film on the back surfaces of the samples (to act as back-electrodes) using e-beam evaporation. The samples were subsequently subjected to annealing at 450 °C in an RTA chamber in an N_2_ atmosphere for 15 min. Photoresist photolithography and lift-off processes were used in conjunction with e-beam evaporation to deposit Ti/Al films (20 nm/500 nm) on the front sides of coatings (to serve as finger-electrodes). Bare solar cells (Figure 1a) measuring 4 × 4 mm^2^ were created via isolation etching using a photolithographic process with a solution of HNO_3_:HF:H_2_O at a ratio of 1:1:2. 

Finally, the front surfaces of the bare solar cells were coated with either a 53-nm-thick indium-tin-oxide (ITO) film (via thermal sputtering) or a 250-nm-thick SiO_2_ film (via spin-on film deposition) (Figure 1b). These films served as antireflection/passivation layers. Specifically, the ITO film was deposited via radio frequency (RF) sputtering (13.56 MHz) at a deposition rate of 0.064 nm/s, a substrate temperature of 260 °C, and an RF power of 45 W. A metallic In/Sn target (90:10 wt%; 2 inch in diameter) with a purity of 99.99% was used as the source of ITO. The silicate solution was applied drop-wise (to clean the bare silicon solar cells) and then held for 10 s before spin-coating was performed at 1200 rpm for 50 s. The deposited sample was then baked at 200 °C for 5 min under an air atmosphere to form a SiO_2_ layer. Optical reflectance (Lambda 35, PerkinElmer, Inc., Waltham, MA, USA), EQE (LiveStrong Optoelectronics Co., Ltd., Kaohsiung, Taiwan), dark current-voltage (I-V) and photovoltaic J-V (XES-151S, San-Ei Electric Co., Ltd., Osaka, Japan) of solar cells ccoated with an anti-reflective layer of SiO_2_ or ITO were measured and compared.

### 2.3. Fabrication And Characterization of Plasmonic Silicon Solar Cells with ITO-NPs Dispersed in SiO_2_ ARC

#### 2.3.1. SiO_2_ ARC with Dispersed ITO Nanoparticles

**Figure 2:**
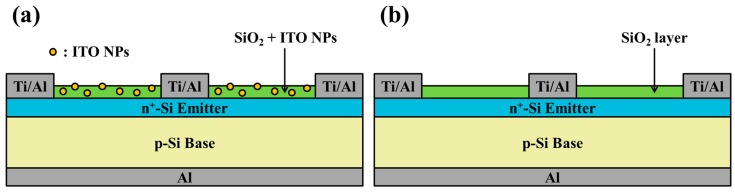
Schematic diagram showing (**a**) a cell with ITO-NPs dispersed in a layer of SiO_2_ (250 nm), (**b**) a cell with a layer of SiO_2_ (250 nm) that did not contain ITO-NPs.

To analyze plasmonic effects, we created cells with two different configurations: (1) Cells coated with a 250-nm-thick SiO_2_ antireflective coating (ARC) that contained dispersed ITO-NPs, and (2) a cell with the same SiO_2_ ARC but without ITO-NPs. In this study, four silicon solar cells were respectively coated with a SiO_2_ ARC containing ITO-NPs at a concentration of 1, 3, 5, or 7 wt% (Figure 2a). As a control, we prepared a solar cell with the same SiO_2_ ARC layer but without ITO-NPs (Figure 2b). The deposition solution comprised 1.94 g of silicate solution combined with ITO powder at a concentration of 1, 3, 5, or 7 wt%. The yellow ITO powder in this study was of 99.99% purity, and the particle sizes were between 20–70 nm. The mixture was applied drop-wise (to clean the bare silicon solar cells) and then held for 10 s before spin-coating was performed at 1200 rpm for 50 s. The deposited sample was then baked at 200 °C for 5 min under an air atmosphere.

#### 2.3.2. Compound ITO/SiO_2_ ARC Comprising ITO Nanoparticles Dispersed in SiO_2_ Layer

**Figure 3:**
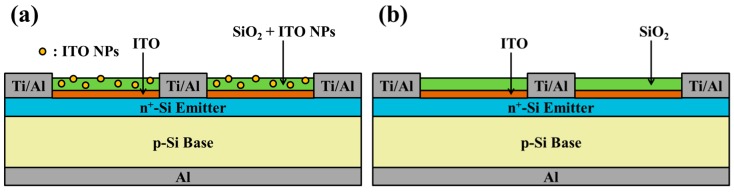
Schematic diagram of (**a**) a silicon solar cell coated with a compound ARC comprising an ITO layer (53 nm) and a layer of SiO_2_ and dispersed ITO-NPs (250 nm); (**b**) a silicon solar cell coated with a compound ARC comprising an ITO layer (54 nm) and a SiO_2_ layer that did not contain ITO-NPs (250 nm).

Four silicon solar cells were respectively coated with a 53-nm-thick ITO layer via thermal sputtering. Over this, a 250-nm-thick layer of SiO_2_ containing ITO-NPs at a concentration of 1, 3, 5, or 7 wt% was deposited via the spin-on film technique. A schematic diagram of the resulting plasmonic silicon solar cell is presented in Figure 3a. As a control, we also prepared a cell with a compound ARC comprising a layer of ITO covered by a layer of SiO_2_ that did not contain ITO-NPs, as shown in Figure 3b. Optical reflectance, EQE, dark I-V and photovoltaic J-V of proposed plasmonic silicon solar cells with compound ITO/SiO_2_ ARC comprising ITO-NPs dispersed in SiO_2_ layer were also measured and compared.

## 3. Results and Discussion 

### 3.1. The Surface Morphology and Optical Properties of the ITO Nanoparticles Dispersed in SiO_2_ Layer

**Figure 4:**
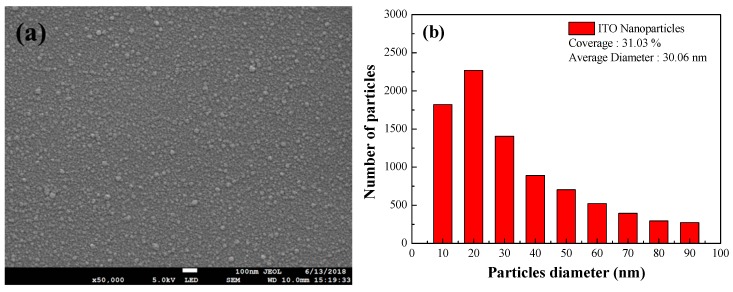
Top-view SEM images of (**a**) 3 wt% ITO NPs dispersed in the SiO_2_ layer; (**b**) size distribution and coverage of 3 wt% ITO NPs, respectively calculated using Image-J software on the SEM image.

**Figure 5:**
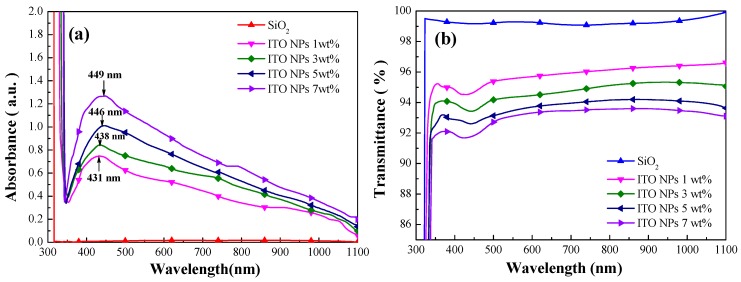
(**a**) The absorbance spectra of ITO NPs dispersed in the SiO_2_ layer at a concentration of 1, 3, 5, or 7 wt%; (**b**) optical transmittance of ITO NPs dispersed in the SiO_2_ layer at a concentration of 1, 3, 5, or 7 wt%.

Figure 4a shows a top-view SEM image of 3 wt% ITO-NPs dispersed in SiO_2_ layer. Figure 4b presents the size distribution and coverage of the ITO particles, which was calculated using Image-J software on the image in Figure 4a. The ITO particles had an average diameter of approximately 30.06 nm and the coverage of approximately 31.03%. The absorbance spectra of ITO-NPs dispersed in SiO_2_ layer at a concentration of 1, 3, 5, or 7 wt% are showed in Figure 5a. The localized surface plasmon resonance (LSPR) absorption peaks of ITO-NPs were located at wavelengths of 431, 438, 446, and 449 nm at the concentration of 1, 3, 5, and 7 wt%, respectively. The absorbance intensity enhanced with the wt% concentrations of ITO-NPs in the full wavelengths. Figure 5b presents optical transmittance of ITO-NPs dispersed in the SiO_2_ layer with various wt% concentrations. The transmittance of the samples with ITO NPs dispersed in SiO_2_ layers was greater than 92% in the full wavelengths. A significant dip exhibited in the transmittance spectrum was attributed to the high LSPR absorption of ITO-NPs. In this study, the absorbance spectrum was in agreement with the optical transmittance for the samples with ITO-NPs dispersed in the SiO_2_ layer at various wt% concentrations.

### 3.2. Characterization of Cells Coated with an Anti-Reflective Layer of SiO_2_ or ITO

**Figure 6:**
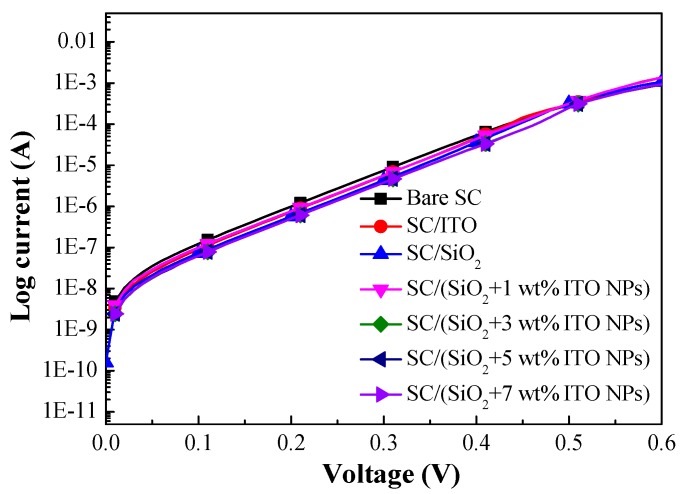
Dark I-V curves of the bare silicon solar cell, a solar cell coated with a SiO_2_ layer (no ITO-NPs), a solar cell with an ITO layer, and solar cells coated with a layer of SiO_2_ containing various concentrations of ITO-NPs.

Figure 6 presents dark I-V curves of a bare silicon solar cell, a cell coated with a layer of SiO_2_, a cell coated with a layer of ITO, and cells coated with a layer of SiO_2_ containing ITO-NPs at a concentration of 1, 3, 5, or 7 wt%. Table 1 lists the ideality factor (*n*) and reverse saturation current (*J_0_*) of all fabricated cells. The *n* and *J_0_* values were as follows: Bare solar cell (1.77 and 6.75 × 10^−9^ A/cm^2^), cell with a SiO_2_ ARC layer but no ITO-NPs (1.73 and 3.62 × 10^−9^ A/cm^2^), cell with an ITO layer (1.74 and 3.18 × 10^−9^ A/cm^2^). Lower *n* and *J_0_* values indicated that the passivation effects of ARC layers suppressed surface recombination. Lower *J_0_* values also indicated enhanced open-circuit voltage (*V_oc_*). Overall, the ITO and SiO_2_ layers presented similar passivation performance.

**Figure 7:**
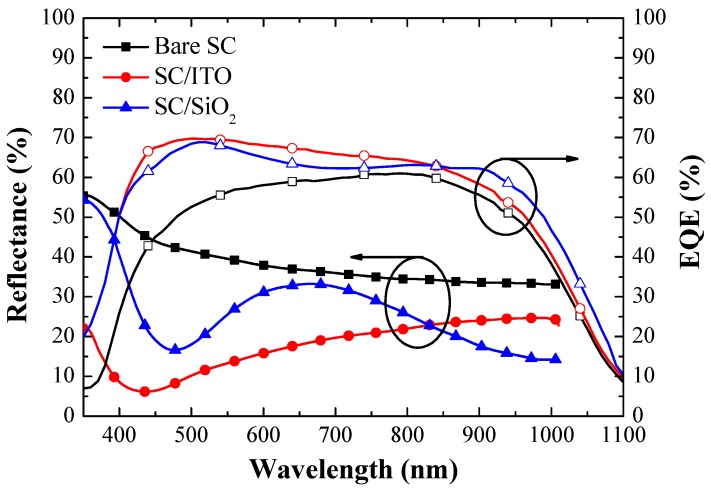
Optical reflectance spectra and external quantum efficiency (EQE) responses of the bare silicon solar cell, the solar cell with a SiO_2_ layer, and the solar cell with an ITO layer.

Figure 7 presents the optical reflectance spectra and EQE responses of the bare silicon solar cell, the cell coated with a layer of SiO_2_ (250 nm), and the cell coated with a layer of ITO (53 nm). The cell with a SiO_2_ layer displayed good anti-reflection characteristics, whereby the lowest values (due to destructive interference) were obtained at a wavelength of approximately 475 nm. The cell coated with an ITO layer also presented good anti-reflection characteristics, with the lowest values obtained at a wavelength of approximately 435 nm. Table 2 lists weighted reflectance (*R_W_*) values calculated over a wavelength range of 350 to 1000 nm. The *R_W_* of the cell coated with ITO (16.48%) was lower than that of the cell coated with SiO_2_ (25.63%) and that of the bare cell (36.75%). Table 2 also lists the weighted EQE (*EQE_W_*) values of all cells, calculated at wavelengths between 350 and 1100 nm. As shown in Figure 7, the SiO_2_ layer clearly enhanced EQE values between 350 and 1100 nm, which was consistent with observed changes in optical reflectance. However, the ITO layer was more effective at enhancing EQE values than the SiO_2_ layer in the wavelength range between 400 and 800 nm, which was also consistent with observed changes in optical reflectance. The overall *EQE_W_* of the cell with an ITO layer (61.60%) also slightly exceeded that of the cell with a SiO_2_ layer (60.45%). In summary, (1) either thermally depositing an ITO layer or spin-on depositing a SiO_2_ layer on silicon solar cells was effective in reducing the effects of reflection; (2) the thermally sputtered ITO film exhibited good electrical and optical performances according to dark I-V, optical reflectance, and EQE results. These findings prompted us to fabricate a solar cell with a compound ARC that comprised an ITO layer beneath a SiO_2_ layer containing ITO-NPs. For the sake of clarity, we calculated the average weighted reflectance (*R_W_*) and the average weighted external quantum efficiency (*EQE_W_*) of the cells as follows:

(1) $$R_{W}=\frac{\int_{350_{}nm}^{1000_{}nm}R(\lambda )\phi_{ph}(\lambda )d\lambda }{\int_{350_{}nm}^{1000_{}nm}\phi_{ph}(\lambda )d\lambda }$$

(2) $$EQE_{W}=\frac{\int_{350_{}nm}^{1100_{}nm}EQE(\lambda )\phi_{ph}(\lambda )d\lambda }{\int_{350_{}nm}^{1100_{}nm}\phi_{ph}(\lambda )d\lambda }$$

where *R(λ)* and *EQE(λ)* are the reflectance and the *EQE* response at a given wavelength (*λ*), respectively, and *φ(λ)* is the photon flux of AM 1.5G at that wavelength (*λ*).

**Figure 8:**
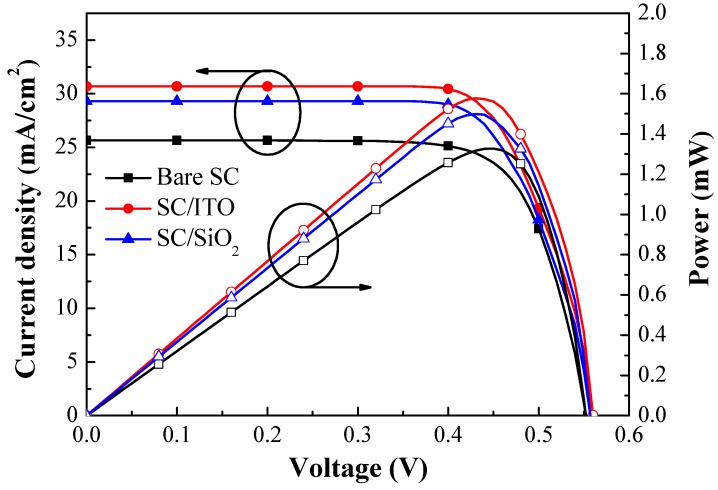
Optical reflectance spectra and external quantum efficiency (EQE) responses of the bare silicon solar cell, the solar cell with a SiO_2_ layer, and the solar cell with an ITO layer.

Figure 8 presents the photovoltaic J-V and power-voltage (P-V) curves obtained from the bare silicon solar cell, the cell coated with a layer of SiO_2_, and the cell coated with a layer of ITO. Table 3 lists the photovoltaic performances of the same cells. The short-circuit current-density (*J_sc_*), open-circuit voltage (*V_oc_*), and conversion efficiency (*η*) values of the bare solar cell were 25.65 mA/cm^2^, 555.01 mV, and 10.67%, respectively. The SiO_2_ ARC layer enhanced the short-circuit current-density (*ΔJ_sc_*) by 14.23% (from 25.65 to 29.30 mA/cm^2^), whereas the ITO layer enhanced *ΔJ_sc_* by 19.61% (from 25.65 to 30.68 mA/cm^2^). The improvements in *J_sc_* that we observed were consistent with observed *EQE_W_* values. In addition, the SiO_2_ layer enhanced conversion efficiency (*Δη*) by 15.27% (from 10.67% to 12.30%), whereas the ITO layer enhanced *Δη* by 19.58% (from 10.67% to 12.76%). The highest output power (*P_max_* = 1.58 mW) was obtained from the cell coated with a layer of ITO. Overall, the ITO layer was superior to the SiO_2_ layer in terms of optical and electrical performance. Thus, we deposited an ITO anti-reflective layer on all silicon solar cells in subsequent experiments. Furthermore, the obtained optical reflectance, EQE and photovoltaic performance were used as a baseline for evaluating the performance enhancement of the cells with ITO-NPs dispersed in the SiO_2_ layer.

### 3.3. Characterization of Silicon Solar Cells Coated with Compound ITO/SiO_2_ ARC Comprising ITO-NPs Dispersed in SiO_2_ Layer

**Figure 9:**
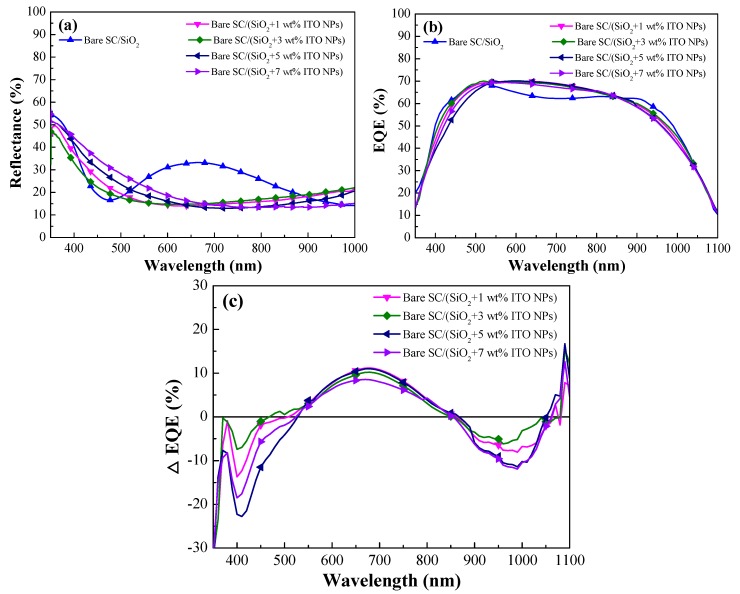
(**a**) Optical reflectance spectra of a cell coated with a layer of SiO_2_ and cell coated with a layer of SiO_2_ containing various concentrations of ITO-NPs; (**b**) external quantum efficiency (EQE) responses of the same cells; (**c**) EQE enhancements (*ΔEQE*) of the same cells, which was compared with the cell with a layer of SiO_2_.

Figure 9a presents the optical reflectance spectra of a solar cell with a SiO_2_ ARC and solar cells coated with a layer of SiO_2_ containing ITO-NPs at a concentration of 1, 3, 5, or 7 wt%. Table 2 lists the *R_W_* values of the same cells. First, the reflectance of cells with a SiO_2_ layer containing ITO-NPs was lower than that of the cell with a simple SiO_2_ layer (without ITO-NPs) at wavelengths between 350 nm and 430 nm, which was attributed to the part of ITO-NPs LSPR absorption. At wavelengths between 550 nm and 900 nm, the reflectance of cells with a SiO_2_ layer containing ITO-NPs was lower than that of the cell with a simple SiO_2_ layer (without ITO-NPs). This could be attributed to (1) destructive interference at the SiO_2_/air and SiO_2_/silicon interfaces as well as (2) forward light scattering caused by the embedded ITO-NPs. The lowest reflectance of cells with a SiO_2_ layer containing ITO-NPs at various wt% concentrations was red-shifted to the wavelength range of 600–800 nm, compared to the cell coated with a layer of SiO_2_. In addition, the lowest reflectance of a layer of SiO_2_ containing ITO-NPs also red shifted to longer wavelengths with an increase in wt% concentration. The increase in reflectance that was observed above 900 nm could be attributed to the backward scattering of incident light by ITO-NPs [22,23,24]. Additionally, the *R_W_* of cells with a SiO_2_ layer containing ITO-NPs (1–7 wt%, as shown in Table 2) was lower than that of the cell with a simple SiO_2_ layer and the lowest *R_W_* was obtained at 3 wt% ITO-NPs in this work because the ITO particles were uniformly dispersed in the SiO_2_ layer at these concentration levels (at > 5 or 7 wt%, some particles aggregated into an island-like piece).

Figure 9b presents EQE responses of a solar cell with a SiO_2_ ARC and solar cells coated with a layer of SiO_2_ ARC containing ITO-NPs at a concentration of 1, 3, 5, or 7 wt%. The *EQE_W_* values of the same cells are listed in Table 2. *EQE_W_* values became greater as the concentration of ITO-NPs increased from 1 wt% to 3 wt%; however, *EQE_W_* values decreased when the concentration of ITO-NPs exceeded 5 wt%. When high concentrations of ITO-NPs were added to SiO_2_ coatings, visible effects were produced. Thus, the decrease in *EQE_W_* values at higher concentrations of ITO-NPs (>5 wt%) can be attributed to the effects of shading and the high reflectivity of small island-like pieces of ITO particles. Overall, the EQE responses of cells with ITO-NPs were in strong agreement with optical reflectance values, and the highest EQE was obtained when the concentration of ITO-NPs was 3 wt%. Furthermore, we investigated the plasmonic effects of ITO-NPs dispersed in SiO_2_ ARC on the cells by EQE enhancements (*ΔEQE*), as shown in Figure 9c. Here, we defined *ΔEQE* of the cells as follows:

(3) $$\Delta EQE={\frac{EQE_{w/ITO-NPs}-EQE_{w/SiO_{2}}}{EQE_{w/SiO_{2}^{}}}}_{}(\%)$$

Negative *ΔEQE* values in the wavelength regions of 350–550 nm and 850–1050 nm are attributed to LSPR absorption and plasmonic backward scattering of incident photons induced by ITO particles, respectively. However, positive *ΔEQE* values in the wavelength regions of 550–850 nm and 1050–1100 nm are suggested for plasmonic forward scattering induced by ITO-NPs dispersed in the SiO_2_ layer.

**Figure 10:**
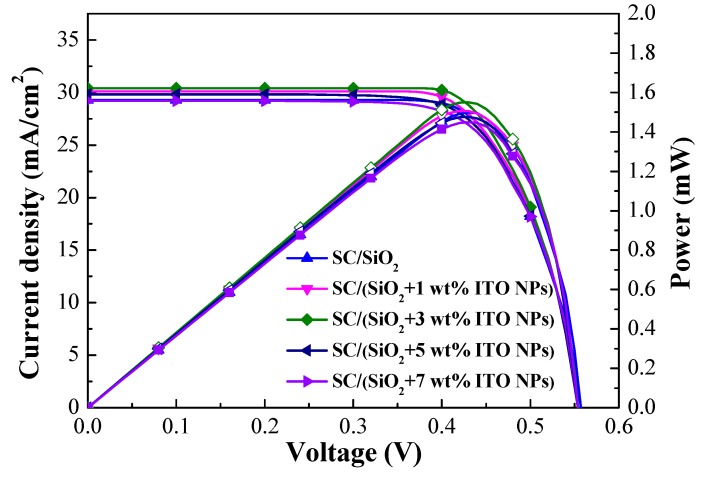
Photovoltaic J–V curves of the cell coated with a layer of SiO_2_ (without ITO-NPs) and cells with a layer of SiO_2_ containing various concentrations of ITO-NPs.

Figure 10 presents photovoltaic J-V curves of the cell coated with a layer of SiO_2_ (without ITO-NPs) and cells coated with a layer of SiO_2_ that contained ITO-NPs at a concentration of 1, 3, 5, or 7 wt%. Table 3 lists the photovoltaic performance of the same cells. The *J_sc_* values were as follows: SC/SiO_2_ without ITO-NPs (29.30 mA/cm^2^), SC/SiO_2_ + 1 wt% ITO-NPs (30.01 mA/cm^2^), SC/SiO_2_ + 3 wt% ITO-NPs (30.41 mA/cm^2^), SC/SiO_2_ + 5 wt% ITO-NPs (29.71 mA/cm^2^), and SC/SiO_2_ + 7 wt% ITO-NPs (29.06 mA/cm^2^). The *η* values were as follows: SC/SiO_2_ without ITO-NPs (12.30%), SC/SiO_2_ + 1 wt% ITO-NPs (12.43%), SC/SiO_2_ + 3 wt% ITO-NPs (12.58%), SC/SiO_2_ + 5 wt% ITO-NPs (12.23%), and SC/SiO_2_ + 7 wt% ITO-NPs (11.90%). The highest *J_sc_* and *η* values were obtained from the cell with 3 wt% ITO-NPs. The highest output power (*P_max_* = 1.55 mW) was obtained from the cell with an ARC of SiO_2_ and 3 wt% of ITO NPs. Based on these results, we employed a layer of SiO_2_ containing 3 wt% ITO-NPs in subsequent experiments for the cells with a compound ITO/SiO_2_ ARC comprising ITO-NPs dispersed in SiO_2_ layer.

**Figure 11:**
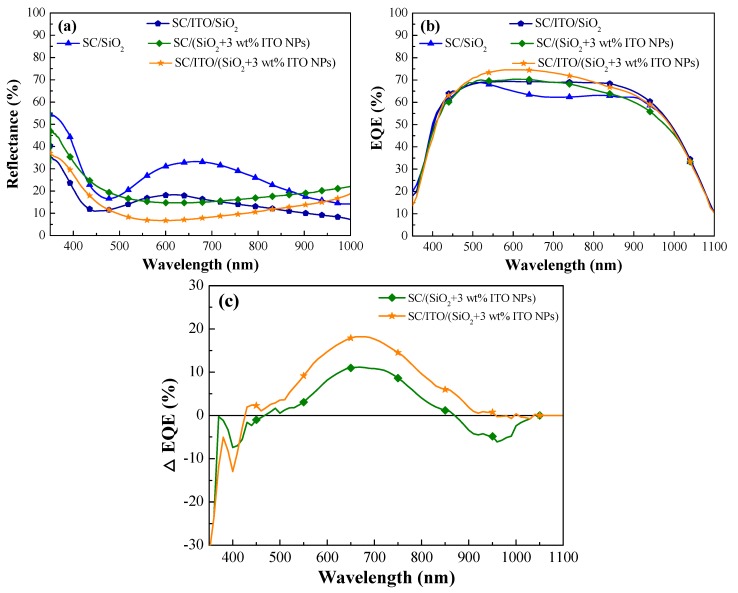
(**a**) Optical reflectance spectra and (**b**) external quantum efficiency (EQE) responses of a cell with an ARC of SiO_2_, a cell with an ARC comprising an ITO layer beneath a SiO_2_ layer, a cell with an ARC of SiO_2_ and 3 wt% ITO-NPs, and a cell with an ARC comprising an ITO layer beneath a layer of SiO_2_ and 3 wt% ITO-NPs. (**c**) *ΔEQE* of a cell with an ARC of SiO_2_ and 3 wt% ITO-NPs and a cell with an ARC comprising an ITO layer beneath a layer of SiO_2_ and 3 wt% ITO-NPs, which was compared with the cell with a layer of SiO_2_ (without ITO-NPs).

Figure 11a presents the optical reflectance spectra of four cells: (1) a cell with an ARC of SiO_2_, (2) a cell with an ARC comprising an ITO layer beneath a SiO_2_ layer (without ITO NPs), (3) a cell with an ARC of SiO_2_ and 3 wt% ITO-NPs, and (4) a cell with an ARC comprising an ITO layer beneath a layer of SiO_2_ and 3 wt% ITO-NPs. Table 2 lists the *R_W_* values of the same cells. The *R_W_* values were as follows: SC/SiO_2_ without ITO-NPs (25.63%), SC/SiO_2_ + 3 wt% ITO-NPs (18.26%), SC/ITO/SiO_2_ without ITO-NPs (14.33%), SC/ITO/SiO_2_ + 3 wt% ITO-NPs (11.41%). In comparing, (1) the *R_W_* value of the cell with ITO/SiO_2_ + 3 wt% ITO-NPs ARC (11.41%) was less than that of 18.26% of the cell with SC/SiO_2_ + 3 wt% ITO-NPs ARC, due to anti-reflection of the ITO layer; (2) the *R_W_* value (11.41%) of the cell with ITO/SiO_2_ + 3 wt% ITO-NPs ARC was less than that of 14.33% of the cell with ITO/SiO_2_ (without ITO-NPs) ARC, due to plasmonic effects of ITO-NPs. Figure 11b presents the EQE responses of the same aforementioned cells and *EQE_W_* values are summarized in Table 2. In summary, the EQE responses of the cells with ITO-NPs were in strong agreement with optical reflectance values. The highest *EQE_W_* value was obtained from the cell with an ARC that comprised an ITO layer beneath a layer of SiO_2_ and 3 wt% ITO-NPs. Moreover, we investigated the plasmonic effects of a single SiO_2_ ARC and compound ITO/SiO_2_ ARC comprising ITO NPs dispersed in a SiO_2_ layer by calculating EQE enhancements, as shown in Figure 11c. Higher Δ*EQE* values were found for the cell with a compound ITO/SiO_2_ ARC than that a single SiO_2_ ARC due to the combined effects of antireflection and plasmonic scattering.

**Figure 12:**
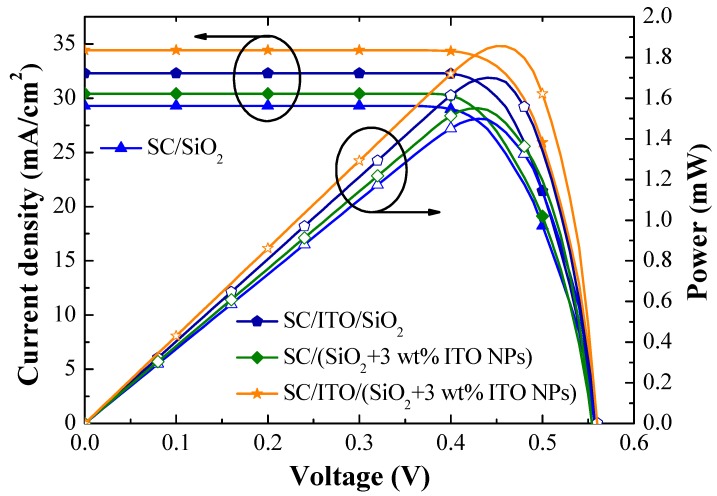
Photovoltaic J-V curves of a cell with an ARC of SiO_2_, a cell with an ARC comprising an ITO layer beneath a SiO_2_ layer, a cell with an ARC of SiO_2_ and 3 wt% ITO-NPs, and a cell with an ARC comprising an ITO layer beneath a layer of SiO_2_ and 3 wt% ITO-NPs.

Figure 12 presents photovoltaic J-V curves of (1) a cell with an ARC of SiO_2_, (2) a cell with an ARC comprising an ITO layer beneath a SiO_2_ layer (no ITO-NPs), (3) a cell with an ARC of SiO_2_ and 3 wt% ITO-NPs, and (4) a cell with an ARC comprising an ITO layer beneath a layer of SiO_2_ and 3 wt% ITO-NPs. Table 3 lists the photovoltaic performance of the same cells. The *J_sc_* and *η* values were as follows: SC/SiO_2_ without ITO-NPs (*J_sc_* = 29.30 mA/cm^2^ and *η* = 12.30%), SC/SiO_2_ + 3 wt% ITO-NPs (*J_sc_* = 30.41 mA/cm^2^ and *η* = 12.58%), SC/ITO/SiO_2_ without ITO-NPs (*J_sc_* = 32.30 mA/cm^2^ and *η* = 13.47%), SC/ITO/SiO_2_ + 3 wt% ITO-NPs (*J_sc_* = 34.43 mA/cm^2^ and η = 14.22%). The significant increase in *J_sc_* and ηvalues could be obtained when ITO NPs were dispersed in SiO_2_. Overall, the cell with an ARC comprising an ITO layer beneath a layer of SiO_2_ and 3 wt% ITO-NPs increased the *J_sc_* by 34.23% and *η* by 33.27%, compared to the bare solar cell, due to the effects of anti-reflection and plasmonic scattering. The highest output power (*P_max_* = 1.86 mW) was also obtained from the cell with an ARC of ITO/SiO_2_ and 3 wt% ITO-NPs.

## 4. Conclusions

In this study, we demonstrated that the thermal deposition of ITO films on silicon solar cells by RF sputtering could greatly reduce reflectance and enhance passivation performance. We also demonstrated that the plasmonic light scattering provided by ITO nanoparticles dispersed in a layer of SiO_2_ could greatly improve the conversion efficiency of silicon solar cells. Compared to a bare (uncoated) silicon solar cell, depositing a layer of SiO_2_ and 3 wt% ITO-NPs improved conversion efficiency by 17.90%, whereas applying that same layer over a sub-layer of ITO improved conversion efficiency by 33.27%, due to the combination of anti-reflection effects and plasmonic scattering. The conversion efficiency of the cell with an ARC comprising an ITO layer beneath a layer of SiO_2_ and 3 wt% ITO-NPs (14.22%) exceeded that of the cell with an ARC layer that only contained SiO_2_ and 3 wt% ITO-NPs (i.e., without the ITO sub-layer) (12.58%).

## Figures and Tables

**Table 1:** Ideality factor (*n*) and reverse saturation current (*J_0_*) of all fabricated cells.

Solar Cell (SC) Structure	*n*	*J_0_* (A/cm^2^) × 10^−9^
Bare SC	1.77	6.75
SC/ITO	1.74	3.18
SC/SiO_2_	1.73	3.62
SC/ITO/SiO_2_	1.73	3.66
SC/(SiO_2_ + 1 wt% ITO-NPs)	1.74	3.97
SC/(SiO_2_ + 3 wt% ITO-NPs)	1.75	4.30
SC/(SiO_2_ + 5 wt% ITO-NPs)	1.75	4.98
SC/(SiO_2_ + 7 wt% ITO-NPs)	1.75	5.2
SC/ITO/(SiO_2_ +1 wt% ITO-NPs)	1.74	3.97
SC/ITO/(SiO_2_ + 3 wt% ITO-NPs)	1.74	4.27
SC/ITO/(SiO_2_ + 5 wt% ITO-NPs)	1.75	4.98
SC/ITO/(SiO_2_ + 7 wt% ITO-NPs)	1.75	5.20

**Table 2:** Weighted reflectance (*R_W_*) and the weighted external quantum efficiency (*EQE_W_*) values of all fabricated cells.

Solar Cell (SC) Structure	*R_W_* (%) (350–1000 nm)	*EQE_W_* (%) (350–1100 nm)
Bare SC	36.75	51.58
SC/ITO	16.48	61.60
SC/SiO_2_	25.63	60.45
SC/ITO/SiO_2_	14.33	63.64
SC/(SiO_2_ + 1 wt% ITO-NPs)	18.75	60.23
SC/(SiO_2_ + 3 wt% ITO-NPs)	18.26	60.98
SC/(SiO_2_ + 5 wt% ITO-NPs)	19.51	60.11
SC/(SiO_2_ + 7 wt% ITO-NPs)	21.01	58.82
SC/ITO/(SiO_2_ + 1 wt% ITO-NPs)	11.86	66.02
SC/ITO/(SiO_2_ + 3 wt% ITO-NPs)	11.41	67.25
SC/ITO/(SiO_2_ + 5 wt% ITO-NPs)	12.10	65.09
SC/ITO/(SiO_2_ + 7 wt% ITO-NPs)	12.67	63.08

**Table 3:** Photovoltaic performance of all fabricated cells.

Solar Cell (SC) Structure	*V_oc_* (mV)	*J_sc_* (mA/cm^2^)	*F.F.* (%)	*η* (%)	*P_max_* (mW)	*ΔJ_sc_* (%)	*Δη* (%)
Bare SC	551.01	25.65	75.51	10.67	1.33	-	-
SC/ITO	560.36	30.68	74.26	12.76	1.58	19.61	19.58
SC/SiO_2_	559.01	29.30	75.08	12.30	1.49	14.23	15.27
SC/ITO/SiO_2_	560.40	32.30	73.98	13.47	1.70	25.92	26.24
SC/(SiO_2_ + 1 wt% ITO-NPs)	554.10	30.01	74.77	12.43	1.51	17.00	16.49
SC/(SiO_2_ + 3 wt% ITO-NPs)	554.01	30.41	74.71	12.58	1.55	18.55	17.90
SC/(SiO_2_ + 5 wt% ITO-NPs)	553.91	29.71	74.33	12.23	1.46	15.82	14.62
SC/(SiO_2_ + 7 wt% ITO-NPs)	553.80	29.06	73.98	11.90	1.45	13.29	11.52
SC/ITO/(SiO_2_ +1 wt% ITO-NPs)	560.59	33.49	73.81	13.86	1.80	30.56	29.89
SC/ITO/(SiO_2_ +3 wt% ITO-NPs)	559.91	34.43	73.76	14.22	1.86	34.23	33.27
SC/ITO/(SiO_2_ +5 wt% ITO-NPs)	559.13	32.84	73.51	13.50	1.73	28.03	26.52
SC/ITO/(SiO_2_ +7 wt% ITO-NPs)	558.86	32.38	73.22	13.25	1.69	26.23	24.17

## References

[1] Araújo A., Mendes M.J., Mateus T., Costa J., Nunes D., Fortunato E., Águas H., Martins R. (2018). Ultra-fast plasmonic back reflectors production for light trapping in thin Si solar cells. Sol. Energy.

[2] Veenkamp R.J., Ye W.N. (2014). Plasmonic metal nanocubes for broadband light absorption enhancement in thin-film a-Si solar cells. J. Appl. Phys..

[3] Beck F.J., Mokkapati S., Catchpole K.R. (2010). Plasmonic light-trapping for Si solar cells using self-assembled, Ag nanoparticles. Prog. Photovolt: Res. Appl..

[4] Catchpole K.R., Polman A. (2008). Design principles for particle plasmon enhanced solar cells. Appl. Phys. Lett..

[5] Atwater H.A., Polman A. (2010). Plasmonics for improved photovoltaic devices. Nat. Mater..

[6] Garcia M.A. (2011). Surface plasmons in metallic nanoparticles: fundamentals and applications. J. Phys. D Appl. Phys..

[7] Kelly K.L., Coronado E., Zhao L.L., Schatz G.C. (2003). The optical properties of metal nanoparticles: the influence of size, shape, and dielectric environment. J. Phys. Chem. B.

[8] Derkacs D., Lim S.H., Matheu P., Mar W., Yu E.T. (2006). Improved performance of amorphous silicon solar cells via scattering from surface plasmon polaritons in nearby metallic nanoparticles. Appl. Phys. Lett..

[9] Pedrueza E., Sancho-Parramon J., Bosch S., Valdés J.L., Martinez-Pastor J.P. (2013). Plasmonic layers based on Au-nanoparticle-doped TiO_2_ for optoelectronics: structural and optical properties. Nanotechnology.

[10] Sharma M., Pudasaini P.R., Ruiz-Zepeda F., Vinogradova E., Ayon A.A. (2014). Plasmonic effects of Au/Ag bimetallic multispiked nanoparticles for photovoltaic applications. ACS Appl. Mater. Interfaces.

[11] Tan H., Santbergen R., Yang G., Smets A.H.M., Zeman M. (2013). Combined optical and electrical design of plasmonic back reflector for high-efficiency thin-film silicon solar cells. IEEE J. Photovolt..

[12] Tong C., Yun J., Song H., Gan Q., Anderson W.A. (2014). Plasmonic-enhanced Si schottky barrier solar cells. Sol. Energy Mater. Sol. Cells.

[13] Lesina A.C., Paternoster G., Mattedi F., Ferrario L., Berini P., Ramunno L., Paris A., Vaccari A., Calliari L. (2015). Modeling and characterization of antireflection coatings with embedded silver nanoparticles for silicon solar cells. Plasmonics.

[14] Manai L., Rezgui B.D., Zaghouani R.B., Barakel D., Torchio P., Palais O., Bessais B. (2016). Tuning of light trapping and surface plasmon resonance in silver nanoparticles/c-Si structures for solar cells. Plasmonics.

[15] Hylton N.P., Li X.F., Giannini V., Lee K.-H., Ekins-Daukes N.J., Loo J., Vercruysse D., Van Dorpe P., Sodabanlu H., Sugiyama M., Maier S.A. (2013). Loss mitigation in plasmonic solar cells: aluminium nanoparticles for broadband photocurrent enhancements in GaAs photodiodes. Sci Rep..

[16] Fantoni A., Fernandes M., Vygranenko Y., Louro P., Vieira M. (2015). Visible range plasmonic effect produced by aluminium nanoparticles embedded in amorphous silicon. Phys. Status Solidi C.

[17] Parashar P.K., Sharma R.P., Komarala V.K. (2017). Mediating broad band light trapping in silicon Solar cell by aluminum nanoparticles with native oxide shell. Mater. Today Proc..

[18] Matsui H., Furuta S., Hasebe T., Tabata H. (2016). Plasmonic-field interactions at nanoparticle interfaces for infrared thermal-shielding applications based on transparent oxide semiconductors. ACS Appl. Mater. Interfaces.

[19] Noginov M.A., Gu L., Livenere J., Zhu G., Pradhan A.K., Mundle R., Bahoura M., Barnakov Y.A., Podolskiy V.A. (2011). Transparent conductive oxides: plasmonic materials for telecom wavelengths. Appl. Phys. Lett..

[20] Sachet E., Losego M.D., Guske J., Franzen S., Maria J.-P. (2013). Mid-infrared surface plasmon resonance in zinc oxide semiconductor thin films. Appl. Phys. Lett..

[21] Kanehara M., Koike H., Yoshinaga T., Teranishi T. (2009). Indium tin oxide nanoparticles with compositionally tunable surface plasmon resonance frequencies in the near-IR region. J. Am. Chem. Soc..

[22] Mandal S., Mitra S., Dhar S., Ghosh H., Banerjee C., Datta S.K., Saha H. (2015). Potential of ITO nanoparticles formed by hydrogen treatment in PECVD for improved performance of back grid contact crystalline silicon solar cell. Appl. Surf. Sci..

[23] Dhar S., Mandal S., Mitra S., Ghosh H., Mukherjee S., Banerjee C., Saha H., Barua A.K. (2017). Light trapping in a-Si/c-Si heterojunction solar cells by embedded ITO nanoparticles at rear surface. J. Phys. D Appl. Phys..

[24] Das G., Mandal S., Dhar S., Bhargav P.B., Banerjee C., Mukhopadhyay S., Barua A.K. (2017). Synthesis of ITO nanoparticles at room temperature using plasma treatment process and use it as back reflector in a-Si flexible solar cell. Surf. Interfaces.

